# Cochrane Library: the best evidence within everyone's reach

**DOI:** 10.1590/1516-3180.2020.138527102020

**Published:** 2020-11-16

**Authors:** Maria Eduarda dos Santos Puga, Álvaro Nagib Atallah

**Affiliations:** I MSc, PhD. Librarian, Evidence-Based Health Program, Universidade Federal de São Paulo (UNIFESP), São Paulo, Brazil; Director, Coordenadoria da Rede de Bibliotecas UNIFESP (CRBU), São Paulo (SP), Brazil. Information Specialist, Cochrane Brazil, São Paulo (SP), Brazil.; II MD, PhD. Full Professor and Head of the Discipline of Emergency Medicine and Evidence-Based Medicine, Universidade Federal de São Paulo (UNIFESP), São Paulo (SP), Brazil. Director of Cochrane Brazil, São Paulo (SP), Brazil.

The Cochrane Library represents excellence in the production of systematic reviews, which are considered to provide the best evidence for diminishing uncertainties within healthcare. It is among the ten most important medical journals worldwide and is available to Brazilians openly and free of charge.^1^^–^^2^

Within the Cochrane Library, there is a collection of databases that provides evidence directed towards Cochrane systematic reviews and their protocols (Cochrane review in progress) and towards Cochrane database systematic reviews (CDSR). The Cochrane Library also includes the largest directory of clinical trials in the world (CENTRAL, the Central Register of Controlled Trials) and Cochrane Clinical Answers, which is an integrated search resource that enables searches in external databases, all within the same tool.^3^

Cochrane reviews are live publications, given that they are updated every two years. The Cochrane Library offers its users the best two levels of evidence for decision-making within healthcare.

## HOW TO ACCESS IT

The web address https://www.cochranelibrary.com/ provides free access to the Cochrane Library. All that is needed is an internet connection.

## HOW TO DO SEARCHES

All production within the Cochrane Library is indexed using the controlled terminology of Medical Subject Headings (MeSH). One useful tip for starting a search is to try to organize it using the acronym **PICO** (**P:** problem/population; **I:** intervention; **C:** control; **O:** outcome). This will be helpful in implementing the search.

Search terms can also be identified through the Portuguese-language official vocabulary of the Descritores em Ciências da Saúde (DECS), which is available from https://decs.bvsalud.org/. From this, the equivalent English-language terms can be copied into each element of **PICO**.

In the Cochrane Library, it is unnecessary to use **T** (type of study) or, as seen in some search-organizing acronyms, **S** (study design), given that searches will find systematic reviews, review protocols and clinical trials. Cochrane searches are already filtered to show these top two levels and syntheses of evidence.

## SIMPLE SEARCH

**Figure 1:**
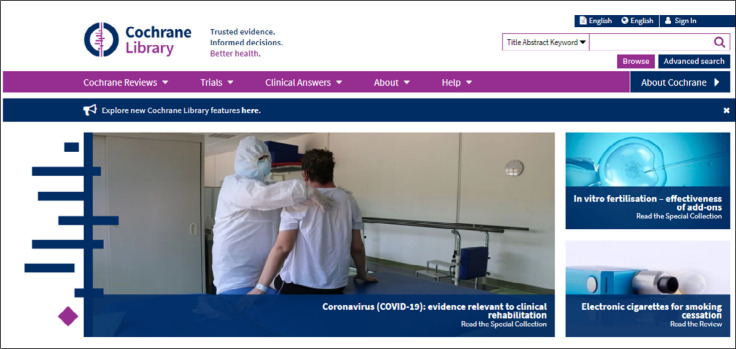
Initial interface for Cochrane search.

**Figure 2:**
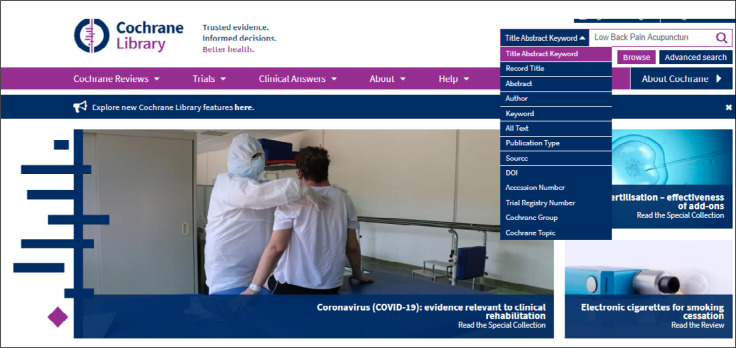
Simple search just using words.

One or more words representing the subject of interest can be entered. The result will identify these words as they appear in article titles, abstracts or keywords (Figure 1). Example: low back pain and acupuncture (P - **Low Back Pain**; I **- Acupuncture**) (Figure 2).

## RESULT FROM SIMPLE SEARCH

**Figure 3:**
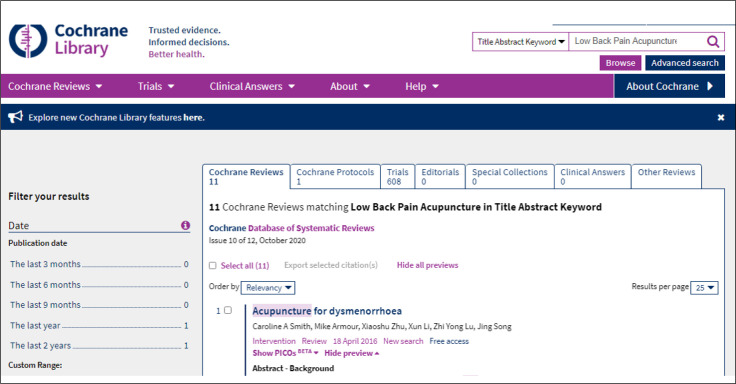
Result from simple search.

The result obtained is presented in terms of directories (filters): Cochrane reviews; followed by Cochrane protocols; reviews registered in the Cochrane database that are in progress; trials, comprising clinical trials gathered in the main databases; and lastly, manual searches through Cochrane centers and groups. These can be viewed by clicking on the different tabs of the results (Figure 3).

## ADVANCED SEARCH

**Figure 4:**
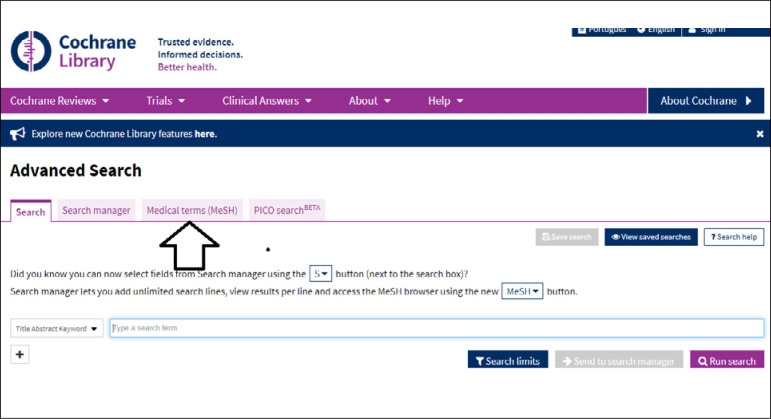
Search using MeSH terms.

**Figure 5:**
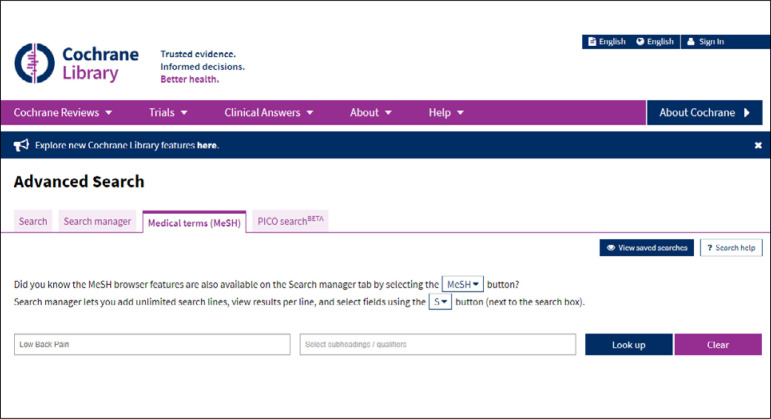
Selection of tab for MeSH terms in advanced search.

The entire Cochrane Library collection is indexed using the MeSH vocabulary. The terms in English can be located via DECS to build up a PICO framework. The Medical Terms (MeSH) tab is then accessed, as indicated in Figure 4, and the term in English can then be entered (Figure 5).

**Figure 6:**
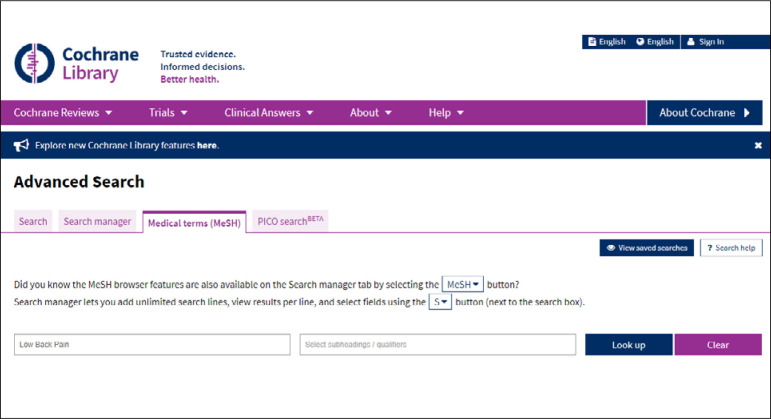
Identification of MeSH terms, which are then loaded into the search strategy.

In the advanced search format, the MeSH term is then located and selected (Figure 6), so that the search will be performed using the official terms. This is done for all the terms used in building up the PICO, by clicking on the **Select** button and then on **Add search manager**. It is important to do this for all the terms in the PICO. The **Search manager tab** is then accessed, which provides the result for each MeSH term identified.

**Figure 7:**
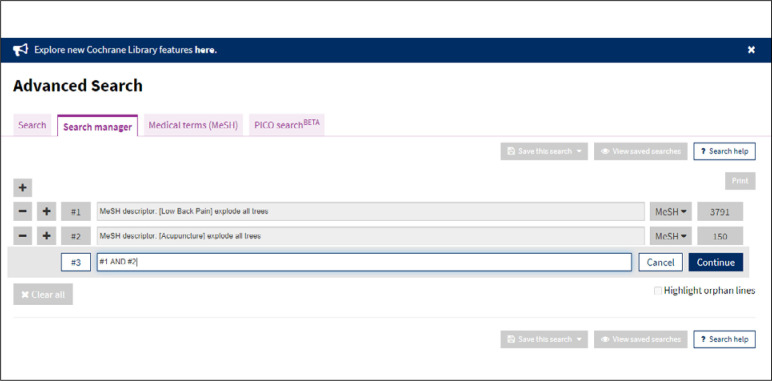
Advanced search strategy.

Intersections between terms can be managed here to obtain the final result. The databases use Boolean operators (OR, AND and NOT). Therefore, in an advanced search strategy, it needs to be specified whether intersections between sets of terms exist (Figure 7).

**Figure 8:**
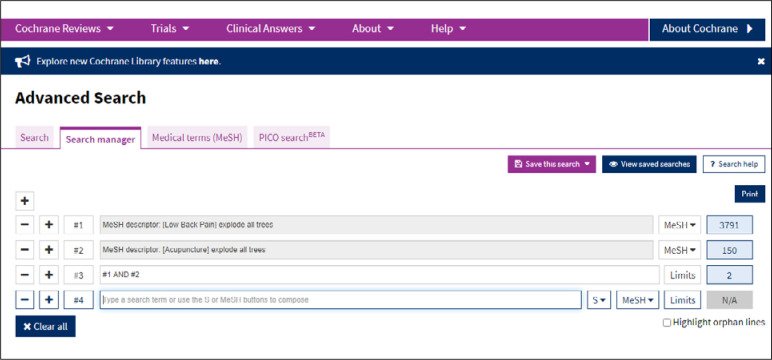
Result from advanced search for each MeSH term.

**Figure 9:**
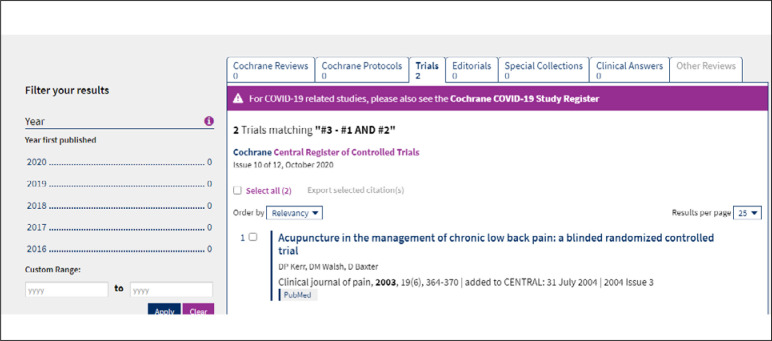
Result from advanced search, with intersection of sets #.

Figure 7 shows the result from each set of terms (#) investigated and also the intersections between sets (#). Set #3 shows an intersection, using AND: #1 AND #2 (Figure 8). By clicking on any of the results, a screen with the data retrieved will appear (Figure 9).

**Figure 10:**
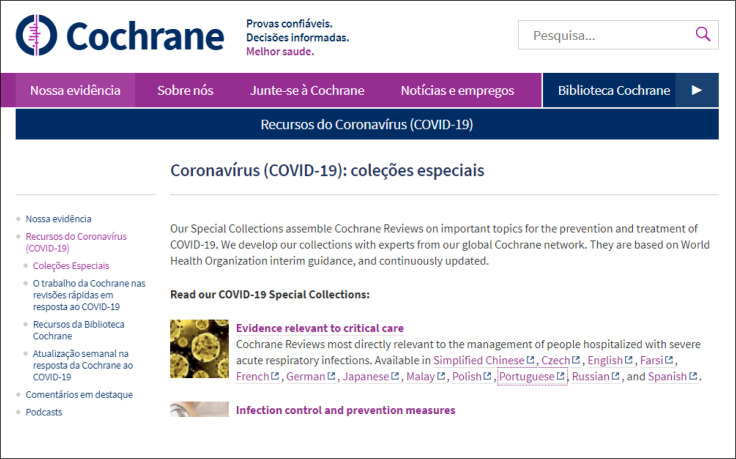
COVID-19 special collection.

**Figure 11:**
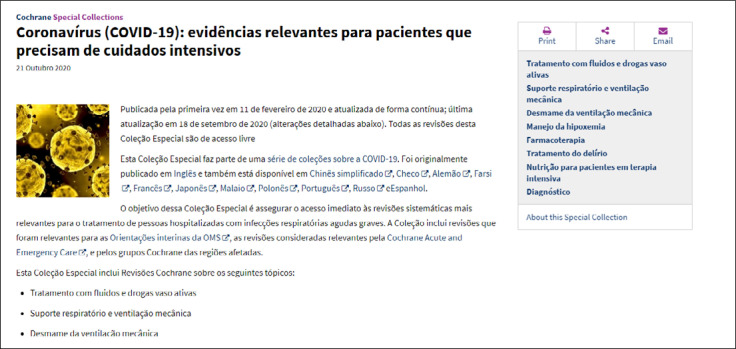
COVID-19 special collection: critical care translated into Portuguese.

The special collections of the Cochrane Library have contributed to publication of a series of robust collections that provide open access to systematic reviews supporting prevention and treatment of COVID-19 (Figure 10). These collections have been translated into several languages, including Portuguese (Figure 11).

The Cochrane Library app is an instrument or resource that allows all healthcare professionals to follow the most recent evidence from Cochrane reviews easily and rapidly. It can also be used for offline reading of the material. Thus, healthcare professionals can create a personal collection of evidence so as to be able to make decisions assertively, from the highest quality of evidence available in the literature.

In Brazil, through funding from the Coordination Office for Improvement of Higher-Education Personnel (Coordenação de Aperfeiçoamento de Pessoal de Nível Superior, CAPES), the Cochrane Library is accessible to everyone, without exception.

The Brazilian Cochrane Center was founded by Dr. Álvaro Nagib Atallah in 1996 and was inaugurated by Dr. Iain Chalmers. It functions as a training center for undergraduate and postgraduate students at the Federal University of São Paulo (Universidade Federal de São Paulo) and other Brazilian universities, and it is open to everyone with an interest in this. It produces systematic reviews for the Cochrane Library, Brazilian Ministry of Health and specialist medical societies, and produces technological assessments of public interest, without conflicts of interest.

## References

[1] Atallah AN (2018). Evidence-based medicine. Sao Paulo Med J.

[2] Atallah AN, Puga MES (2020). Web of Science Journal Citation Report 2020: the Brazilian contribution to the “Medicine, General & Internal” category of the journal impact factor (JIF) ranking (SCI 2019). Sao Paulo Med J.

[3] Cochrane Library Trusted evidence. Informed decisions. Better health.

